# Use of weighted Fourier linear combiner filters to estimate lower trunk 3D orientation from gyroscope sensors data

**DOI:** 10.1186/1743-0003-10-29

**Published:** 2013-03-11

**Authors:** Vincent Bonnet, Claudia Mazzà, John McCamley, Aurelio Cappozzo

**Affiliations:** 1 LABLAB, Department of Human Movement and Sports Sciences, University of Rome “Foro Italico”, Rome, Italy

## Abstract

**Background:**

The present study aimed at devising a data processing procedure that provides an optimal estimation of the 3-D instantaneous orientation of an inertial measurement unit (IMU). This result is usually obtained by fusing the data measured with accelerometers, gyroscopes, and magnetometers. Nevertheless, issues related to compensation of integration errors and high sensitivity of these devices to magnetic disturbances call for different solutions. In this study, a method based on the use of gyroscope data only is presented, which uses a Weighted Fourier Linear Combiner adaptive filter to perform a drift-free estimate of the 3D orientation of an IMU located on the lower trunk during walking.

**Methods:**

A tuning of the algorithm parameters and a sensitivity analysis to its initial conditions was performed using treadmill walking data from 3 healthy subjects. The accuracy of the method was then assessed using data collected from 15 young healthy subjects during treadmill walking at variable speeds and comparing the pitch, roll, and yaw angles estimated from the gyroscopes data to those obtained with a stereophotogrammetric system. Root mean square (*RMS*) difference and correlation coefficients (*r*) were used for this purpose.

**Results:**

An optimal set of values of the algorithm parameters was established. At all the observed speeds, and also in all the various sub-phases, the investigated angles were all estimated to within an average *RMS* difference of less than 1.2 deg and an average *r* greater than 0.90.

**Conclusions:**

This study proved the effectiveness of the Weighted Fourier Linear Combiner method in accurately reconstructing the 3D orientation of an IMU located on the lower trunk of a subject during treadmill walking. This method is expected to also perform satisfactorily for overground walking data and to be applicable also to other “quasi-periodic” tasks, such as squatting, rowing, running, or swimming.

## Background

Inertial measurement units (IMUs) have gained in popularity as a tool to quantify human motion [1], thanks to their ease-of-use, robust design, and their small dimensions. In the same time, electronics mass-market companies provided low cost devices, such as cell-phones, that contain embedded IMU devices along with recording and transmission capabilities. These advantages enable their use for extended periods outside the confines of a laboratory.

An IMU normally includes accelerometers and gyroscopes to measure three accelerations and three angular velocities, respectively. Theoretically, the determination of the position and orientation in space of such a device could be obtained by double and single integration of the above signals, respectively. Unfortunately, the IMU outputs are subject to drift over time, which jeopardizes the time integration of the raw signals [2]. The additional use of magnetometers has been proposed to compensate for the integration errors, but their effectiveness is limited by their high sensitivity to magnetic disturbances [3,4].

The present study focused on the estimate of orientation and aimed at devising a data processing procedure that would compensate for the above-mentioned drift and provide an optimal estimation of the 3-D instantaneous orientation of the IMU and, therefore, of the body segment it is attached to.

As reported in the literature, the use of recursive filters, such as the Kalman filter, allows the real-time accurate estimate of lower trunk 2D orientation (pitch and roll angles) from the measurement of three accelerations and three angular velocities [5]. A possible alternative is represented by model-based adaptive filters, which can be used when it is possible to formulate reliable hypotheses about the shape and the time evolution of the signal, which is plausible when the type of motor task performed is known [2]. Among the most popular model-based adaptive filters are the Fourier Linear Combiner (FLC) filters [6,7]. These filters, which model the measured signal by a Fourier series, are effective when dealing with periodic signals, which is hardly ever the case in biomechanics in general, and in human movement in particular. For this reason, while dealing with the real-time analysis and cancellation movements such as hand tremor, Riviere and his colleagues [8] proposed the use of Weighted Fourier Linear Combiner (WFLC) filters. These filters are an extension of the FLC to be used when investigating signals that display an oscillatory pattern but with a time-varying period. Human walking is a phenomenon that, although for analysis purposes is most of the time hypothesized to be periodic, it does exhibit minor features that vary from stride to stride and, as such, may be defined as quasi-periodic. WFLC filters could hence be considered to be suitable for analyzing gait related data.

This paper proposes the use of a WFLC adaptive filter to perform drift-free 3D orientation angle estimation starting from the measurement of three angular velocities as provided by three orthogonally mounted gyroscopes. The accuracy of the method was assessed using data concerning the orientation of the lower trunk and collected while volunteers walked on a treadmill at different speeds, both at quasi steady-state and in accelerating and decelerating conditions.

## Methods

The proposed method for the estimate of the 3D sensor orientation is based on two main steps. First, a tracking of each of the three measured angular velocity components is performed by identifying the corresponding Fourier series coefficients using the WFLC. The identified Fourier series are then analytically integrated to estimate the three orientation angles.

### The WFLC filter

**Figure 1:**
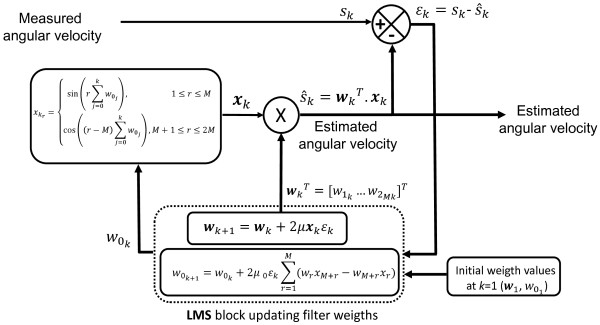
**Block diagram of the weighted fourier linear combiner filter. **Block diagram of the weighted fourier linear combiner filter.

As previously mentioned, the WFLC is an adaptive filter that allows the analytical tracking of a quasi-periodic signal. The architecture of the WFLC is presented in Figure 1. The input of the WFLC is the angular velocity signal as measured at the instant of time *k* (*s*_*k*_). Depending on the instantaneous difference *ε*_*k*_ between the signal *s*_*k*_ and the output estimated by the WFLC, $\left({\hat{S}}_{k}\right)$, the WFLC computes the Fourier series coefficients that will represent the measured signal at time *k*+1. This result is obtained by adjusting, at each iteration, the so-called filter weights, $w_{0_{k}}$ (the frequency weight, taking into account for the fundamental pulsation) and ***w***_*k*_ (the vector containing the amplitude weights), using the least mean square (LMS) algorithm proposed by Widrow et al. [9]. Initial values of the weights, $w_{0_{1}}$ and ***w***_1,_ need, of course, to be provided.

The state vector $x_{k}={\left[x_{1_{k}}\ldots x_{2M_{k}}\right]}^{T}$ used by the WFLC is composed of the sine and cosine functions computed using the frequency weight $w_{0_{k}}$[6-8]:

(1a) $$x_{r_{k}}=\{\begin{array}{l} sin\left(r\sum_{j=0}^{k}w_{0_{j}}\right),\;1\;\underline{<}\;r\;\underline{<}\;M\\ cos\left(\left(r-M\right)\sum_{j=0}^{k}w_{0_{j}}\right),M+1\underline{<}\;r\underline{<}\;2M \end{array}$$

where *M* is the order of the Fourier series representing the measured signal *s*.

At each instant of time, $w_{0_{k}}$ and $w={\left[w_{1_{k}}\ldots w_{2M_{k}}\right]}^{T}$ are computed using the following equations:

(1b) $$\varepsilon_{k}=s_{k}=w_{k}^{T}x_{k}-w_{b_{k}}$$

(1c) $$w_{0_{k+1}}=w_{0_{k}}+2\mu_{0}\varepsilon_{k}\sum_{r=1}^{M}m\left(w_{r}x_{M+r}-w_{M+r}x_{r}\right)$$

(1d) $$w_{k+1}=w_{k}+2\mu x_{k}\varepsilon_{k}$$

with *μ*_0_ and *μ* being the so called frequency and amplitude adaptation gains, respectively. $w_{b_{k}}$ is introduced in the computation of *ε*_*k*_ to estimate the bias present in the signal [9-11], due to possible low frequency components and/or drift, and remove it. It is computed as:

(1e) $$w_{b_{k+1}}=w_{b_{k}}+2\mu_{b}\varepsilon_{k}$$

where *μ*_*b*_ is the so called bias adaptation gain.

The three adaptation gains, *μ*_0_, *μ,* and *μ*_*b*_ determine the convergence time, the accuracy of the algorithm in tracking the measured signal, and the algorithm stability at each sample of time. It has to be noted that the algorithm convergence time cannot be analytically computed [8] and that high values of the gains can improve tracking of the input signal, but can also cause the algorithm to diverge.

In order to allow the use of higher gains, the WFLC can be run twice [8]: the first time using high *μ*_0_ and low *μ* values to identify the frequency weight $w_{0_{k}}$ and the second time using the so identified $w_{0_{k}}$ and a higher *μ* (*μ*_*FLC*_) to identify ***w***_k_. It has to be noted that the latter use of the WFLC algorithm, in which equation 1c is ignored, makes it equivalent to an FLC [6-8].

### Analytical integration

The estimates of the IMU orientation require an integration of the gyroscopes’ data. Once the angular velocity is tracked with the above algorithms, its analytical and instantaneous representation is provided by the identified Fourier series. The analytical integration of this Fourier series removes drift issues, and can be computed at each sampled instant of time using the following method [2], [11,12]: first the vector $w_{i_{k}}={\left[w_{i_{1_{k}}}\ldots w_{i_{2M_{k}}}\right]}^{T}$ is computed using the identified amplitude, and frequency weights:

(2) $$w_{i_{r_{k}}}=\{\begin{array}{l} -w_{r_{k}}/\left(rw_{0_{k}}f_{s}\right),\;1\underline{<}\;r\;\underline{<}\;M\\ -w_{r_{k}}/\left(\left(r-M\right)w_{0_{k}}f_{s}\right),\;M+1\;\underline{<}\;r\;\underline{<}\;2M \end{array}$$

where *f*_s_ is the sampling frequency. The instantaneous estimate ${\hat{s}}_{i_{k}}$ of the integral of the measured angular velocity is then obtained as [2], [11,12]:

(3) $${\hat{s}}_{i_{k}}=w_{i_{k}}{}^{T}.x_{k}$$

**Figure 2:**
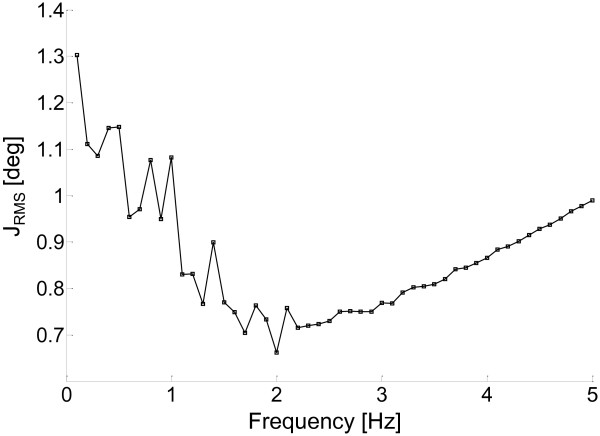
**Results of the sensitivity analysis: initial frequency weight. **The figure shows the *J*_*RMS *_values plotted as a function of the observed range of initial frequency weight values (*w*_0_).

The above angle represents the result of the integration of each single angular velocity component along its corresponding sensor axis, which does not necessarily coincide with the actual rotation of the corresponding axis of the IMU local frame (Figure 2). To overcome this issue, an estimate of the actual rotation can be obtained through a rigid transformation that accounts for the fact that at every instant of time there is a rotation of the local system of reference (x, y, z) fixed to the IMU with respect to the one computed at the previous instant of time.

### Experimental session

A total of 18 volunteers (10 males, 8 females, age range: 24–64 years, stature: 1.76±0.09 m, mass: 78±11 kg) were included in the study after signing an informed consent. They were asked to perform three walking trials at natural walking speed (as measured over level ground), 80% and 120% of natural speed on a motorized treadmill. The subjects initially stood on the treadmill, which was then accelerated to the desired velocity. After 35 s of steady state walking, the treadmill was decelerated and stopped for 5 s, before being reaccelerated to the same velocity for additional 35 s and then stopped again, for a total recording time of 80 s. The transition and stopping phases were used to assess the ability of the proposed algorithm to provide accurate estimates also during non-periodic motion over a short interval of time.

An IMU (Freesense, Sensorize srl) was located on the lower back of the subjects so that the unit local frame (ULF) axes were aligned with the anatomical axes of the lower trunk. In addition, three retro-reflective markers were attached to the IMU sensor in order to define a marker-cluster local frame (MLF). Acceleration and angular velocity data were collected from the IMU (*f*_*s*_*=*100 samples·s^-1^) while the marker trajectories were tracked by five infrared cameras (MX, Vicon, *f*_*s*_*=*100 samples·s^-1^).

Pitch, roll and yaw angles, describing the orientation of the ULF, were estimated from the IMU data using the WFLC algorithm and those describing the orientation of the MLF were reconstructed using the stereophotogrammetric system. The time invariant offset of the MLF orientation relative to the ULF orientation was mathematically removed through a rigid transformation while the subject was standing still. In this way both instruments could be assumed to provide pitch, roll, and yaw angles of the same lower trunk anatomical frame. Pitch, roll and yaw angles, can hence be associated with lower-trunk frontal and lateral bending and axial rotation, respectively.

It should be noted that in this study the stereophotogrammetric errors [13] propagate to the angles of interest causing a maximal inaccuracy of 0.5 deg.

### Tuning of the algorithm parameters

The order of the Fourier series *M,* representing the measured signal *s,* was set to *M*=1. This conservative choice was initially made following the literature [2,8] and was supported by a preliminary analysis, in which higher values of *M* caused convergence problems, leading to very inaccurate and unrealistic estimates for some of the investigated trials. Conversely, the choice of *M*=1 guaranteed the stability of the algorithm for all trials and all subjects.

In order to provide the best trade-off between accuracy, stability and robustness of the above described method, data recorded from three randomly selected subjects at three different speeds were used to determine the optimal combination of the WFLC gains *μ*, *μ*_*0*_, *μ*_*b*_. To this purpose, the three combinations of gains that minimize the three cost functions defined for the pitch, roll, and yaw angles, *J*_*P*_, *J*_*R,*_*J*_*Y*_*,* respectively, were searched for. Each of these cost functions was calculated as the average over the nine trials of the root mean square (*RMS*) differences between the angle calculated from the stereophotogrammetric data and the one calculated from the IMU.

Knowing that all the gains need to be positive, noting that previous implementations of the FLC algorithm suggest that in most cases a value of μ<0.5 is most likely to ensure convergence [6,7], and on the basis of the authors experience, the following range of values were considered in the gain identification process:

$$\begin{array}{l} \mu =0.01+i0.025,\;i=1,2,3,\ldots 10\\ \mu_{\mathrm{FLC}}=5\mu \\ \mu_{0}=\mu_{b}=\left[5{\text{e}}^{-8};\;1{\text{e}}^{-7};\;5{\text{e}}^{-7};\;1{\text{e}}^{-6};\;5{\text{e}}^{-6};\;1{\text{e}}^{-5};\right)\\ \;\left(5{\text{e}}^{-5};\;1{\text{e}}^{-4};\;5{\text{e}}^{-4};\;1{\text{e}}^{-3}\right] \end{array}$$

The WFLC algorithm requires an initial estimate of the value of the frequency weight *w*_*0*_ (equation 1c and Figure 1). A sensitivity analysis of the effects of varying *w*_*0*_ was performed to choose this value by searching for the frequency value that minimized the cost function *J*_*RMS*_ = (*J*_*P*_ + *J*_*Y*_ + *J*_*R*_)/3. The tested range of frequencies was 0.1-5 Hz, which is expected to include all possible walking related frequencies.

### Assessment of estimate accuracy

**Figure 3:**
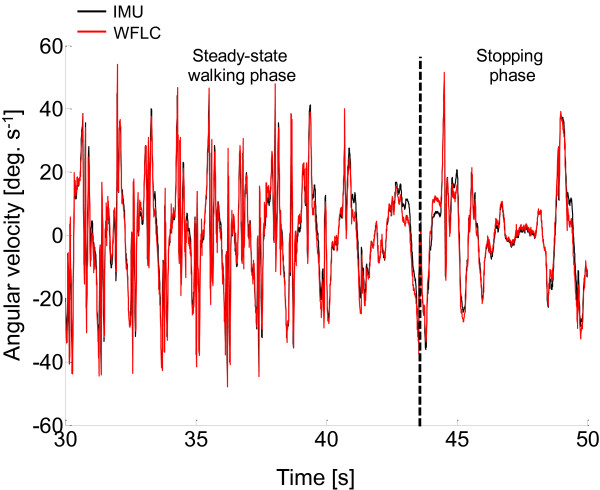
**Angular velocity tracking. **Representative results of one walking trial showing the estimated (red line) and measured (black line) angular velocity components.

The accuracy of the WFLC in the estimate of the pitch, roll, and yaw angles was assessed by comparing them to the corresponding angles obtained from the stereophotogrammetric data in the 45 trials that were not used in the gains identification process. The *RMS*, correlation coefficient *r*, and offset values between estimated and measured angles were calculated for the whole trials (80 s), for the steady-state sub-phase (25 s), and for the transient sub-phase, which included the deceleration, stopping and acceleration phases (15 s) (Figure 3).

## Results

**Figure 4:**
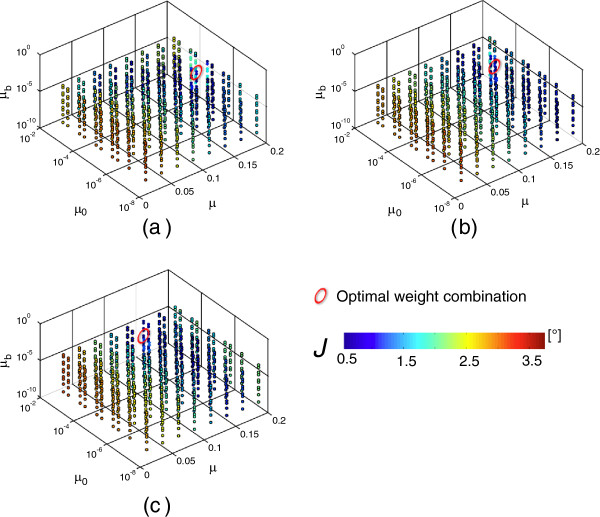
**Results of the sensitivity analysis: weight parameters. **Results of the gains identification process: values of *J*_*P *_for pitch **(a)**, *J*_*R *_for roll **(b)**, and *J*_*Y *_for yaw **(c) **angles are shown. Data have been plotted for the combinations of the 3 parameters *μ*, *μ*_*0*_, *μ*_*b*_, with the corresponding *J *values represented using a color scale. The red ellipses indicate the optimum weight combinations.

Results of the gain identification procedure are shown in Figure 4. The red circles indicate the optimum weight combination for pitch (a); roll (b), and yaw (c) angles, respectively. The optimum gain values, which will be used on all the following computations, were: Pitch: *μ*=0.160, *μ*_*0*_=1e^-5^, *μ*_*b*_=5e^-4^; Roll: *μ*=0.160, *μ*_*0*_=5e^-5^, *μ*_*b*_=1e^-3^; Yaw: *μ*=0.135, *μ*_*0*_=1e^-3^, *μ*_*b*_=5e^-4^. The corresponding *RMS* values were *J*_*P*_=0.8±0.2 deg, *J*_*R*_=0.4±0.1 deg, and *J*_*Y*_=1.0±0.5 deg.

The results of the sensitivity analysis concerning the choice of *w*_*0*_ are shown in Figure 2. The values obtained for *J*_*RMS*_ ranged from 1.3 to 0.65 deg, with very small variations observed for *w*_*0*_ varying in the range of 1–3 Hz, indicating a low sensitivity of the outputs to this initial condition.

The minimum *J*_*RMS*_ value was found for *w*_*0*_*=*2 Hz*.*

The ability of the WFLC algorithms to track a measured angular velocity is depicted in Figure 3, where the results are shown for one randomly selected entire trial. It should be noted that, after the stopping phase, the algorithm rapidly re-converges as soon as the subject starts walking again.

**Figure 5:**
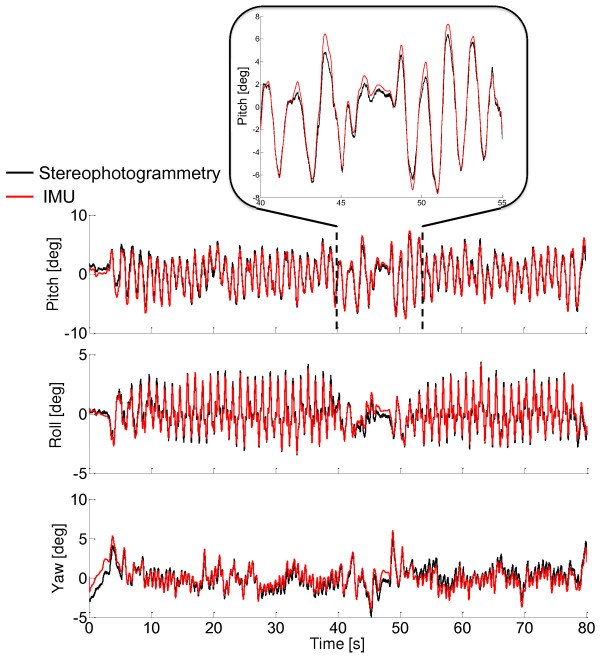
**Representative trunk angles estimate. **Representative results obtained for one randomly selected trial. The lower trunk orientation angles, as obtained from the stereophotogrammetric system (black curves) and using the proposed method (red curves) are shown. Angles are expressed in the ULF.

As shown in Figure 5 for one randomly selected trial, the proposed algorithm led to very satisfactory results in terms of accuracy for the pitch, roll, and yaw angles, not only in the steady-state walking phase (from 10 s to 35 s), but also during the decelerating (from 40 s to 42 s), stopping (from 42 s to 45 s), and accelerating (from 45 s to 48 s) phases (Table 1).

**Table 1:** Results of the accuracy analysis

		**Pitch**			**Roll**			**Yaw**		
	**Trials**	***RMS *(deg)**	***r***	**Offset (deg)**	***RMS *(deg)**	***r***	**Offset (deg)**	***RMS *(deg)**	***r***	**Offset (deg)**
Whole trial	Slow	0.9±0.3	0.93±0.04	2.2±1.6	0.5±0.1	0.92±0.04	0.7±0.4	1.1±0.6	0.80±0.16	2.4±3.0
	Natural	0.8±0.2	0.95±0.03	1.4±1.4	0.4±0.1	0.94±0.02	0.9±0.0	1.1±0.7	0.82±0.14	2.6±2.6
	Fast	0.9±0.3	0.93±0.03	2.4±1.5	0.4±0.0	0.95±0.02	0.9±0.0	1.2±0.7	0.82±0.19	2.9±2.8
Steady state	Slow	0.7±0.2	0.97±0.02	2.0±1.6	0.4±0.1	0.95±0.03	0.8±0.4	0.7±0.4	0.86±0.14	2.9±2.9
	Natural	0.8±0.2	0.96±0.02	1.4±1.4	0.4±0.1	0.97±0.02	0.9±0.0	1.1±0.7	0.83±0.17	2.6±2.6
	Fast	0.7±0.3	0.96±0.03	2.7±1.7	0.4±0.1	0.96±0.05	0.7±0.6	1.1±0.8	0.86±0.24	2.9±2.4
Stopping Phase	Slow	0.6±0.2	0.98±0.01	2.4±1.9	0.4±0.1	0.96±0.02	0.8±0.7	0.7±0.4	0.93±0.04	2.9±3.4
	Natural	0.8±0.2	0.98±0.02	2.4±1.4	0.4±0.1	0.95±0.01	0.9±0.0	1.1±0.7	0.94±0.03	2.6±2.6
	Fast	0.7±0.2	0.98±0.01	2.3±1.5	0.4±0.1	0.96±0.03	0.7±0.0	1.2±0.8	0.89±0.06	2.9±3.1

The mean (standard deviation), *RMS*, *r* and offset values for the 45 trials that were not used for the gains identification are reported in Table 1. At all the observed speeds, all the investigated angles were estimated within an average of less than 1.2 deg and with average correlation coefficients greater than 0.90 (with the highest values found for the yaw angles). This applied to both the whole trials and to their sub-phases. An average offset of less than 3 deg was found, with lowest values observed for the roll angle.

## Discussion

The aim of this study was to validate a method based on the use of a WFLC adaptive filter approach, to obtain a drift-free estimate of the 3D orientation of a sensor attached to the lower trunk for a prolonged period of time during treadmill walking, from angular velocities recorded using only one IMU.

A tuning of the WFLC was initially performed, to find optimal values of its gains. A sensitivity analysis was then performed to assess the effects of changes in algorithm frequency weight *w*_*0*_, which is crucial for ensuring that equation 2, and hence the output of the proposed method, are not determinate. Results of this analysis showed that the outputs were always determinate for frequencies ranging between 0.1 Hz and 5 Hz, and that frequencies ranging from 1 Hz to 3 Hz led to very similar results. It has to be noted that these frequencies are actually those expected to be of interest when dealing with human locomotor tasks.

After the above tuning process, the method proved to be very accurate in estimating all the three angles, for all the observed speed conditions and also when the subjects were not walking at steady state. Interestingly, the convergence time of the algorithms, which generally depends on the signal properties, appeared to be negligible for the specific investigated application, as shown by the fact that the results obtained for the transition phases were almost identical to those obtained in the steady state phase (Table 1). This ability of the method to provide accurate angle estimations during non-periodic motion (acceleration and deceleration phases) and during short intervals of almost no motion (stopping phase) opens the way to future applications, such as uncontrolled walking.

The accuracy of the estimates of lower trunk bending in the sagittal (pitch) and frontal (roll) planes is similar to that obtained in a previous study using a properly optimized Kalman filter [5]. A clear advantage of the proposed method is that, conversely from the previous approach, it uses only the angular velocity signals. Nevertheless, the Kalman filter approach is expected to be more robust for non-periodic motions than the proposed method, since it does not require any a-priori assumption about the signal characteristics.

It has been previously shown that when tracking signals that have a frequency content composed by many frequencies that are close to each other, the performance of the WFLC can be degraded [8]. A Band-limited Multiple Fourier Linear Combiner (BMFLC) [12], [14] can be used to overcome this problem. However, the BMFLC filter requires an *a priori* determined set of frequencies, which is not always available when dealing with human movement analysis applications [15,16]. Numerical integration, as associated with WFLC-BMFLC adaptive filters, has been recently used successfully for tremor cancelation [14]. This numerical approach, however, requires the use of a high-pass filter, which allows easy separation of the tremor oscillations (high frequency) from the voluntary motion (low frequency). Unfortunately, this approach is not suitable for lower trunk angular velocity data recorded during walking, when the determination of high-pass filter cut-off frequency is not straight forward due to the variability of walking speed and to the fact that most of the gyroscope signals power is within the low frequencies, which hinder the determination of a proper high-pass cut-off frequency

In conclusion, this study proved the effectiveness of the WFLC method in accurately reconstructing the 3D orientation of an IMU located on the lower trunk of a subject during treadmill walking. This method is expected to also perform satisfactorily for overground walking data. The small differences in the values of the measured angular velocities which might be observed between treadmill and level walking data, might require an adjustment of the identified values of the algorithm gains. Further studies are needed to test the suitability of the method for the assessment of pathological gaits and to examine the generalizability of the method to other “quasi-periodic” tasks, such as squatting, rowing, running, or swimming.

## Competing interests

The authors declare that they have no competing interests.

## Authors’ contributions

VB implemented the algorithms, processed the data and contributed to the drafting of the manuscript. CM designed the study, supervised the experimental sessions and contributed to the drafting of the manuscript. JM carried out the experimental sessions and contributed to the drafting of the manuscript. AC contributed to the design and drafting of the manuscript. All authors read and approved the final manuscript.
